# Bag of Visual Words Model with Deep Spatial Features for Geographical Scene Classification

**DOI:** 10.1155/2017/5169675

**Published:** 2017-06-19

**Authors:** Jiangfan Feng, Yuanyuan Liu, Lin Wu

**Affiliations:** College of Computer Science and Technology, Chongqing University of Posts and Telecommunications, Chongqing 400065, China

## Abstract

With the popular use of geotagging images, more and more research efforts have been placed on geographical scene classification. In geographical scene classification, valid spatial feature selection can significantly boost the final performance. Bag of visual words (BoVW) can do well in selecting feature in geographical scene classification; nevertheless, it works effectively only if the provided feature extractor is well-matched. In this paper, we use convolutional neural networks (CNNs) for optimizing proposed feature extractor, so that it can learn more suitable visual vocabularies from the geotagging images. Our approach achieves better performance than BoVW as a tool for geographical scene classification, respectively, in three datasets which contain a variety of scene categories.

## 1. Introduction

Geographical scene classification can effectively facilitate the environmental management, indoor and outdoor mapping, and other analysis in dealing with spatial data. Spatial features have been proven to be useful in improving the representation of the image and increasing classification accuracies. In practice, a widely used method named bag of visual words (BoVW) finds the collection of local spatial features in the images and combining appearance and spatial information of images. The traditional BoVW model tends to require the identification of the spatial features of each pixel with a small number of training samples. However, despite the fact that their advantages have been proven in small data sets, their performance is variable and less satisfactory while dealing with large datasets due to the complexity and diversity of landscape and land cover pattern. Therefore, it is a challenge to obtain higher interpretation accuracies when dealing with increased geographical imagery.

To overcome these drawbacks, we combined the CNN-based spatial features and the BoVW-based image interpretation into geographical scene classification. The convolutional neural network (CNN) is a biologically inspired trainable architecture that can learn multilevel hierarchies of features, which perfectly meet the demand of feature learning tasks. With multiple layers, the input images' geographical features can be extracted and generated hierarchically and then expressed as geotags. Owing to the special architecture of CNN, the features learned by the networks are invariant to translation, scaling, tilting, and other forms of distortion of input images, reducing the influence of the deformation of the same geologic structure.

In detail, we utilize the invariance property of BoVW model. At the same time, the methods of preprocessing the geographic scene were taken, and the adaptability of the network structure was modified, so as to improve the generalization ability of the classification model. We combine the CNN-based feature extractor with the BoVW-based scene classification method in this study. The use of the CNN here helps us to look for the appropriate spatial feature extractors for BoVW more adaptively and methodically. The spatial features are obtained by training CNN of the original data set. The strategy of combining the BoVW with CNN-based feature extractor can well reveal the intrinsic properties of original data. And the experimental results show that the proposed method is more invariant to various transformations, which produces a much higher level representation of richer information and achieves superior performance at the geographic scene classification.

## 2. Related Works

Local feature descriptor has been widely used in the scene classification task, as it is a fundamental element for the low-level image representation. Over the past decade, considerable efforts have been devoted to designing appropriate feature descriptors for scene classification. As early as 2004, Csurka and other scholars [1] first formally proposed a visual word packet model algorithm for image scene classification. Good performance is achieved by using the local features in scene classification algorithm [2, 3]. Later Lazebnik et al. [4] proposed SPM (spatial pyramid matching) based on SIFT features, and the SIFT features in the scene classification had achieved a good performance. The GIST features proposed by Oliva and Tarralba [5] are to capture the spatial structure characteristics of the scene and ignore the subtle texture information of the objects or backgrounds contained in the image. In addition to using the label category level, the algorithm based on local feature can also use the bounding box information of the image [6]. These partial image features can be viewed as an expansion of image features from the lower to mid-level semantic features; what is more, these mid-level features can be obtained with more information of visual elements [7].

However, when the image type reaches over thousands of categories and the database capacity exceeds one million, the huge amount of data usually becomes difficult for the traditional method based on low-level features and high-level semantics; fortunately, the deep learning method based on large data has a better performance. In particular, the deep convolutional neural network has made a new breakthrough in scene classification tasks. CNNs are multilayer classes of deep learning models that use a single neural network to train the end-to-end pixel values from the original image to the output of classifier. Compared with the traditional neural network, CNN is more suitable for displaying the spatial structure of the image. Convolution corresponds to the local features of the image and pooling makes the feature obtained by convolution with translation invariance. The idea of CNNs was firstly introduced in [8], improved in [9], and refined and simplified in [10, 11]. The latest state of the art in image classification is the GoogLeNet, a deep CNN which has 22 layers [12]. Convolutional neural networks are very effective at learning image representations with shift-invariant features, directly from an original input image without any handmade feature extraction [13]. Thus, the application of convolutional neural network is more extensive, and its powerful ability of feature recognition has been fully reflected. The pretraining of CNNs in large databases (such as ImageNet) can be used as a universal feature extractor, which can be applied to other visual recognition tasks, and is far better than the method of manual feature design [14–18]. For larger datasets with very high spatial and spectral resolution, the deep learning frameworks seem to be suitable and more efficient in solving hyperspectral image classification [19–21]. In order to reduce the gap between naive feature extraction and methods relying on domain expertise, Vakalopoulou et al. [22] present an automated feature extraction scheme based on deep learning. And by integrating additionally spectral information during the training procedure, the calculated deep features can account for the building to not-building object discrimination [23]. Furthermore, Zuo et al. [18] propose a deep learning based approach that encodes pixels spectral and spatial information for hyperspectral data classification.

Although the chain structure of CNN had been used to solve the general rough classification problem, it did not do well in the geographic scene classification. The global representation of the coded representation is invariant to various transformations, but it has lost the spatial information. The representation of convolutional feature is more discriminative than the fully connected feature [24], and it is more sensitive to translation, rotation, and scalation [25]. In recent years, some traditional methods have been introduced into deep learning models, such as SVM, which is applied to CNN, and the CNN-SVM method [26] is proposed. Compared with the original model, the classification performance is improved, and it is proved that SVM as a classifier of depth model is very feasible. He et al. [27] found that in depth training convolutional neural network needs a uniform size and uniform size of image zoom will cause image or cutting deformation; they made the space of pyramid (Spatial pyramid Pooling, SPP polymerization). The aggregation layer can unify the output characteristics of different sizes to a uniform size, which is convenient to connect to the final fully connection layer. Cimpoi et al. [24] used the pretrained CNN on the PLACES database to extract the MIT-67 database of the convolutional feature and the whole connection feature and then used the linear SVM for classification. It turned out that the BoVW, VLAD, and Fisher Vectors coding method based on the convolutional feature can effectively improve the global representation of the image. However, in the study of An et al. [10], BoVW is on the invariance to image translation, rotation, and zoom, but the dictionary in the BoVW model is unsupervised learning (such as *k*-means) and it is unable to obtain the optimal dictionary adaptive classification task. BoVW model of unsupervised learning (such as *k*-means clustering) is relatively independent with convolutional features of supervised learning, and it could not obtain the optimal dictionary for adaptive classification tasks. Generally speaking, our method develops the hierarchical feature learning structure, which gets superior performance at the geographic scene classification.

## 3. Proposed Approach

As discussed above, geographical scene classification aims at categorizing images according to spatial semantic property. In the view of geographical scene classification, the semantics of images are relationships and difference among different geographical features. Generally speaking, our method develops the multilevel hierarchical feature learning structure, which gets superior performance at the geographical scene classification.

### 3.1. Overview

**Figure 1:**
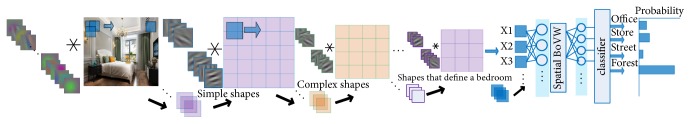
The architecture of our model.

As is shown in Figure 1, we firstly use convolutional neural networks (CNNs) for optimizing proposed feature extractor to get more information from the geotagging images and then choose an appropriate layer to be embedded in BoVW model to learn more suitable visual vocabularies, finally getting result by training a classifier.

The most straightforward way of increasing the performance of a neural network is to increase the depth and width of the network. However, many drawbacks are produced out with this method. For example, the larger the network is, the more parameters it contains, which make the network easier to overfit. Also, the use of computational resources is expanded dramatically. As a result, the fundamental approach of solving both issues would be by ultimately moving from fully connected to sparsely connected architectures, even inside the convolutions. As described in the next section, we train a network model that is suitable for extracting features.

### 3.2. Feature Extraction

The network structure of convolutional neural networks compared with the traditional network has three main points, which are locally connected, shared weights, and subsampling. The receptive field, the kernel, the number of layers, and the number of feature maps of each layer are primary variables affecting the quality of deep features. In this paper, we study how to extract the geographic features with various spatial correlations automatically and find out the visual structure information with automatic recognition in the image and focus on the feature extraction of the image content according to the identified region. We input the characteristics into the visual word bag model to achieve efficient identification of geographical images.

In the convolutional layer, we assume that the given large original image *l* × *h* is defined as *x*. At first, we obtain the small size image *a* × *b* from the large-size image by training the sparse coding. And we obtain *k* characteristics by calculating *f* = *σ*(*wx*_*s*_ + *b*), and *σ* is an activation function; *w* and *b* are the weights and deviations between the visual layer unit and the implicit unit. For each small image, we get the corresponding value *f*_*s*_ = *σ*(*w*^1^*x*_*s*_ + *b*^1^), the convolution values of these *f*_*s*_, and the matrix of convolution of the characteristics.

After obtaining the characteristics by convolution, these characteristics need to be classified. In theory, people can use all the extracted features to train the classifier, but too many parameters will make the training of the classifier difficult and prone to overfitting. Selecting the characteristics of different locations for polymerization statistics is called pooling. So we select the maximum pool or the average pool.

### 3.3. The Combination of BoVW Model and CNN

In order to learn the representation end-to-end, we design a CNN architecture that simulates this standard classification channel in a unified and principled manner with different modules. Given a geographical image, we crop the CNN at the last convolutional layer and view it as a dense descriptor extractor. That is to say, the output of the last convolutional layer is a *H* × *W* × *F* map which can be considered as a set of *F*-dimensional descriptors extracted at *H* × *W* spatial locations. Among them, *W* and *H* represent the width and height of the feature map, respectively. *F* represents the number of channels, that is, the number of feature maps. Since each image contains a set of low-dimensional feature vectors, which has similar structure as dense SIFT, we propose to encode these feature vectors into a single feature vector using standard BoVW encoding. Namely, we design a new pooling layer inspired by the spatial bag of visual words that pools extracted descriptors into a fixed image representation and its parameters are learnable via back-propagation.

In this paper, we use the dictionary as a convolution dictionary *D*. Whether the convolutional dictionary *D* = [*d*_1_, *d*_2_,…, *d*_*k*_]can be supervised or not depends on the encoding mode of the convolutional vectors. Each encoding coefficient in the coding vector *C*_*I*_ = [*C*_*i*1_, *C*_*i*2_,…, *C*_*ik*_] must be a compound function about the convolutional words *d*_*k*_ and the convolutional vector *F*_*i*_, and the *F*_*i*_ model parameters can be guided. Here we use the soft distribution coding method; formula (1) is as follows:

(1) $$\begin{array}{c} c_{ik}=\frac{\exp\left(-\beta {\left\|F_{i}-d_{k}\right\|}_{2}^{2}\right)}{\sum_{k=1}^{K}\exp\left(-\beta {\left\|F_{i}-d_{k}\right\|}_{2}^{2}\right)}. \end{array}$$

The encoding coefficients *c*_*ik*_ represent the degree of membership of the convolutional vector and the convolutional words. During the model training, we found that the soft distribution coding coefficients *c*_*ik*_ tend to zero or saturation, and it leads to the gradient disappearance of the model and makes the model unable to train. Therefore, in this paper, a method based on weighted direction similarity is used in the experiment. It uses a positive value of the vector dot product to indicate that it can be viewed as a weighted directional similarity, such as formula (2):

(2) $$\begin{array}{c} c_{ik}={\left[\;\langle \;F_{i},d_{k}\;\rangle \;\right]}_{+}. \end{array}$$

Among them, [·]_+_ is said to take a positive part, and the negative part returns to zero. From the point of view of the activation function, [·]_+_ is similar as ReLU function, which is a kind of nonsaturation nonlinear transformation function. After the end of encoding direction similarity, all encoding vector volume laminated polymerized (i.e., the global summation of the spatial dimension and the pooling) image global representation. The maxout activation function is used to carry out nonlinear transformation to the adjacent dimension of the representation, and the response is selected to form the final BoVW representation. In this paper, the BoVW representation is used to replace the full link representation *P* = [*p*_1_, *p*_2_,…, *p*_*k*/2_], and the enhancement model is invariant to various image transformations. As illustrated in Figure 1 the layer can be visualized as a metalayer that is further decomposed into basic CNN layers connected up in a graph.

### 3.4. Image Representation Based on Spatial Vision

Csurka et al. [1] first proposed the BoVW model of Natural Language Processing field into image classification. Similar to the document consisting of a number of words, each image is represented as a visual word frequency histogram, in order to achieve better classification results. Bag of visual words model requires an unsupervised algorithm to extract low-level features from the images of the training image set, such as *K*-means algorithm, and clusters these low-level features according to the number of given cluster centers.

**Figure 2:**
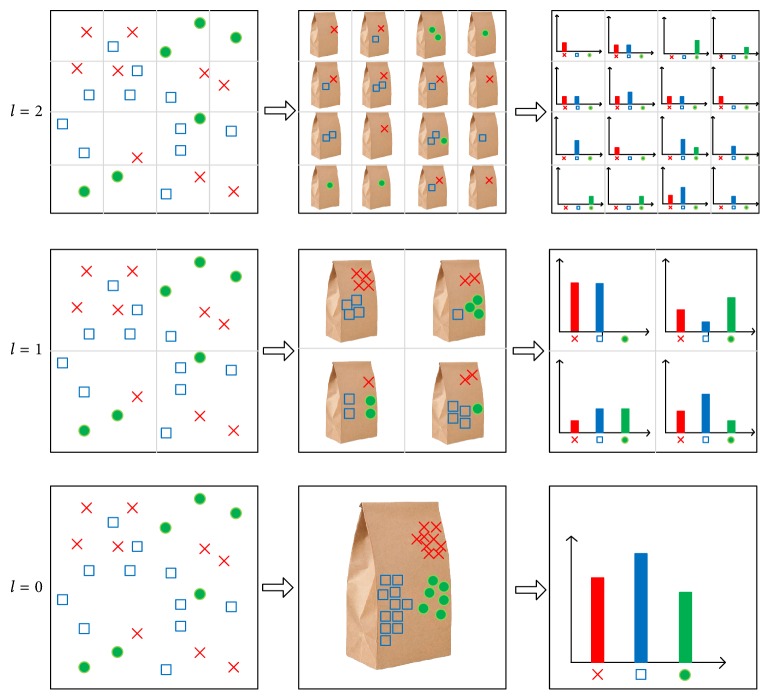
Space pyramid visual word bag model.

Because the traditional bag of words model does not consider the visual spatial information between local features, and it ignores the position order information of the image features, which is not conducive to the image feature extraction space. So Lazebnik et al. [4] put forward visual spatial pyramid bag of words model to make up for deficiencies. The idea of the spatial pyramid is to divide the images in different scales on each scale. Each image subregion is represented as a histogram vector, and the vectors of all subregions on each scale are combined to form a whole. The spatial pyramid histogram vector gives the image of the space of the gold tower vector representation. It repeats the provision of increasingly fine mesh sequences in the feature space to form a multiresolution histogram pyramid. Then the similarity between the two feature sets is defined as the weighted sum of the feature matching numbers at each level of the pyramid. And the visual spatial pyramid bag of words model is shown in Figure 2.

**Figure 3:**
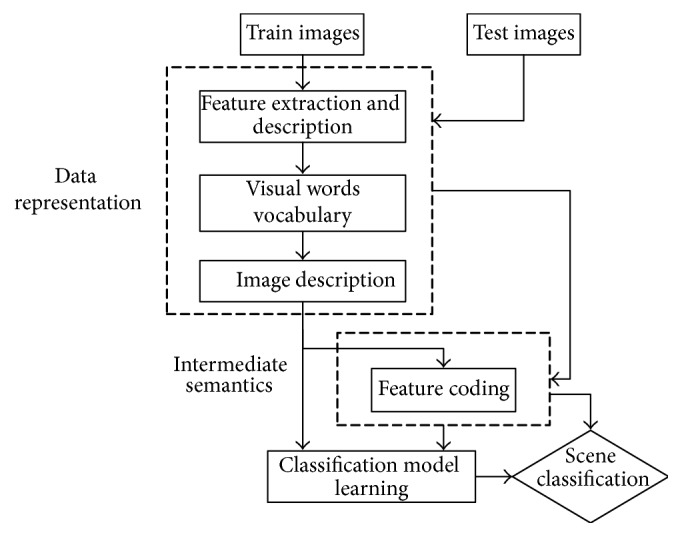
Overview of spatial BoVW model in scene classification.

Scene classification is to extract the image features of all kinds of scenes from the scene image database and then classify all kinds of scenes. The basic content of the scene image classification includes data representation, classification model learning, and judgment. And it has a branch in the middle added semantic analysis topics. Overview of spatial BoVW model in scene classification block diagram as shown in Figure 3.

## 4. Experiments and Results

In this section, we first describe the experimental data sets and image preprocessing method. Afterwards, we describe CNN configuration and the parameter settings of the proposed method. The results obtained for the scene classification are last discussed.

### 4.1. Description of Data Sets

**Figure 4:**
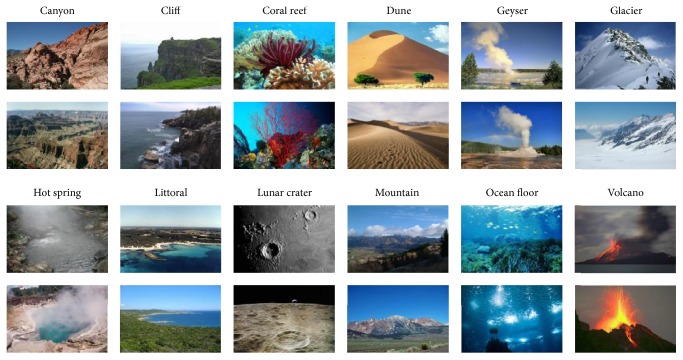
A few example in twelve-scene categories set.

Here, we examine the proposed approach with two popular datasets and one our own dataset.  Figure 4 shows a few example images representing various scenes that are included in the dataset we made, and the contents of the three data sets we choose are summarized here.

15-scene data set is composed of fifteen-scene categories: thirteen were provided by Li and Perona [28] (eight of these were originally collected by Oliva and Torralba [5]), and two (industrial and store) were collected by Lazebnik et al. [4]. This scene database is the most widely used database in the scene classification task, and it is appropriate enough (15 classes and approximately 10K images) to enable running a large number of experiments in a reasonable amount of time. Within each scenario are challenges relevant to complex topographies, unique spatial pattern, and different structures inside the region, and so forth.

Most geographical scene classification methods worked well for outdoor scenes but perform poorly in the indoor domain. The MIT indoor 67 [29] contains 67 scene categories and a total of 15,620 images, such as bakery, library, gym, and meeting room, and so forth. And the subtle classification of these interior layouts poses a great challenge to the sensitivity of the method in the geospatial pattern.

And the data set we made is based on ImageNet dataset, including twelve classes. ImageNet is an image database organized according to the WordNet hierarchy, where each node hierarchy is depicted by thousands of images. These images are collected from the web and manually annotated by artificial use of Amazon's Turkish robots. Geographic images are one of ImageNet's 12 semantic branches. There are 175 subclasses under the branch, some of which contain more than 500 images, and some do not contain any image. For a better training network, we select 12 categories for experimentation, each of which contains more than 1000 images. This dataset contains a changing geographical appearance and is realistic about the needs of the geographical scene classification. A few examples in twelve-scene categories set are as shown in Figure 4.

In order to generate a large amount of training data which are similar to the original data in real time, we use the random combination of affine transformations (translation, rotation, scaling, and cropping), horizontal inversion, and elastic cutting to amplify the training data. These methods can reduce the overfitting of model training and make the model get better generalization performance.

### 4.2. Preprocessing

For the input image, we adopt two preprocessing methods, including histogram averaging and Laplacian sharpening. Histogram averaging can increase the smoothness of the image, so that the image will not appear bright or dark situation. Since Laplacian is a differential operator, its application enhances the gray-scale region of the image and attenuates the slowly changing region of gray.

**Figure 5:**
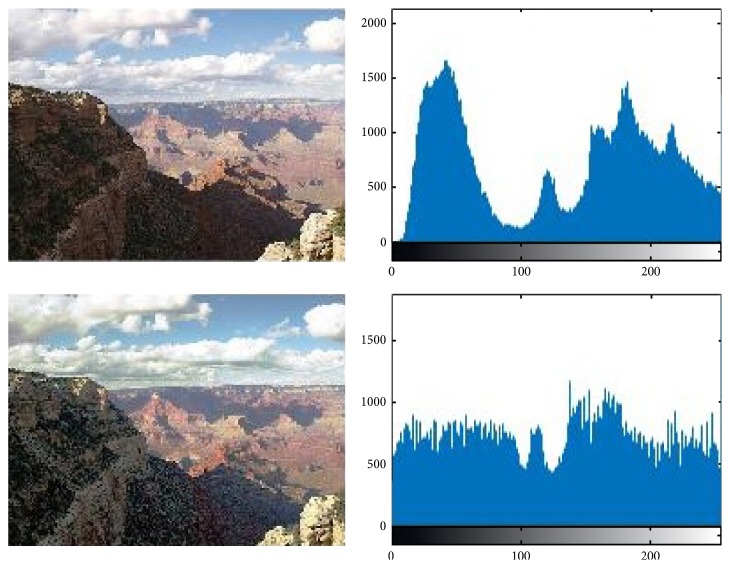
An example image before and after being processed with the average filter.

**Figure 6:**
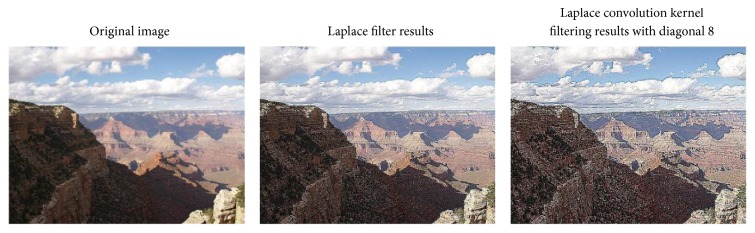
An example image before and after applying Laplacian operator.

Figures 5 and 6, respectively, intuitively display that the contrast of the picture changes. Through the contrast figures above, we can intuitively see that the image enhancement based on histogram equalization and specification can reduce light noise of the captured image and improve the contrast details and the dynamic range of gray level of the image.

**Figure 7:**
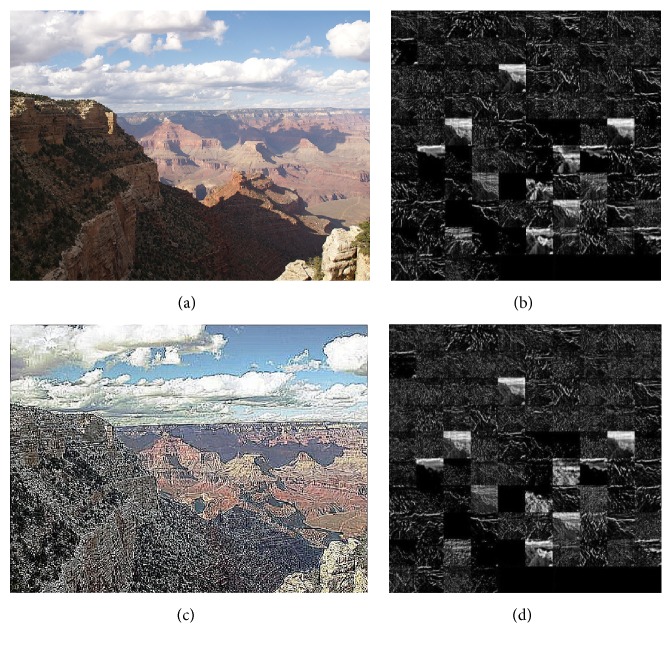
A comparison of the visualization of features before and after pretreatment.

In order to verify the effect of image preprocessing on image feature representation, we randomly select a picture and enter a model that has been trained. The second convolutional layer feature graph is visualized after the input network of the preprocessed picture, and the second layer feature map after the input network of the unprocessed picture is also visualized. And we compare these two visual images in Figure 7.

As shown in Figure 7, we explain a little bit; (a) is the original picture; (b) is the visualization of the feature map of the second layer of the original image; (c) shows the picture after the preprocessing operation, and we can observe that the spatial structure of the picture is clearer; (d) graph is the visualization of the feature of the second layer after the image input network is preprocessed. By comparison we empirically found that taking the following two steps as a preprocession helps to improve the image regularization without much trade-off for the feature reconstruction error.

### 4.3. CNN Configuration

#### 4.3.1. Network Architecture

In this paper, we choose deep learning frameworks named TensorFlow [35] to create and train our CNN model to extract the feature. During the deep feature extraction process, it is important to address the configuration of the deep learning framework. We will draw a vivid image to show the architecture of the our proposed CNN and introduce the network in detail.

**Figure 8:**
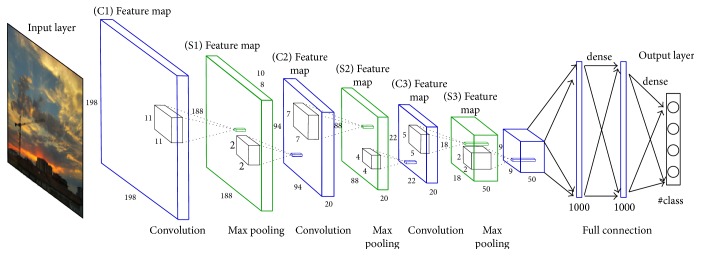
The architecture of the proposed CNN.

Figure 8 shows our network architecture that can be summarized as 198 × 198-94 × 94 × 20-22 × 22 × 20-9 × 9 × 50-1000-1000-*M*, where *M* is the number of classes. The input is a down sampled and normalized image of size 198 × 198. The network model of feature extraction we used has eight layers; three layers are convolutional layers, with each layer of laminated roll being pool layer and last two layers are fully connected layers. We empirically set the size of the receptive field to 11 × 11, which offered enough contextual information. Two fully connected layers of 1000 nodes each follow the convolutional and pooling layers. The last layer is a logistic regression with softmax that outputs the probability on each class, as defined in the following equation:

(3) $$\begin{array}{c} P\left(\;y=i\;|\;x,W_{1},\ldots ,W_{M},b_{1},\ldots ,b_{M}\;\right)=\frac{e^{W_{i}x+b_{i}}}{\sum_{j=1}^{M}e^{W_{j}x+b_{j}}}, \end{array}$$

where *x* is the output of the second fully connected layer, *W*_*i*_ and *b*_*i*_ are the weights and biases of the neuron in this layer, and *M* is the number of classes. All weights are initialized to 10^−2^ and biases are set to 0. The class that outputs the max probability is taken as the predicted class, which can be described in the following equation ($\hat{y}$ denotes the predicted class):

(4) $$\begin{array}{c} \hat{y}=arg\;\underset{i}{max}\;P\left(\;y=i\;|\;x,W_{1},\ldots ,W_{M},b_{1},\ldots ,b_{M}\;\right). \end{array}$$

The receptive field is a term originally to describe an area of the body surface where a stimulus could elicit a reflex [36]. From the CNN visualization point of view, the receptive field is the output feature map node response corresponding to the input image area. In the training process, the cross entropy loss value unifies the normalized prediction value and the normalized coding of the label. TensorFlow provides a visualization tool TensorBoard which can be used to visualize changes in cross entropy during training.

In order to obtain the suitable scale characteristics in the case of a given database, it is necessary to find a suitable granularity. To further verify the effect of receptive field transforms, we conducted the experiments in 15-scene. We constantly adjust the size of the field according to the value of loss and classification results. We find that the transformation of the receptive field, in order to get more accurately modeling of complex invariances, does lead to a nontrivial increase in accuracy gain. One might speculate that using larger receptive fields could replace our receptive field transforms. The reasoning would be that larger receptive fields, hence larger neighborhoods, could be sufficient in capturing the change in position of features under complex view conditions. We repeated the experiment with a setup in which the receptive fields of the pooling grids in the final layer and the receptive field transform functions are set to be an identity function. The result was a decrease in accuracy over the base system which confirms our earlier claim that traditional pooling fails to meet requirement of complex invariance at object feature level and that the problem is not solvable by choice of receptive field size.

**Figure 9:**
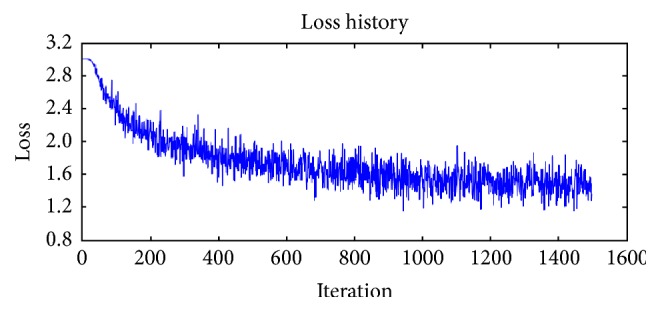
Training loss of our baseline network.

Figure 9 illustrates regression loss decrease against number of iterations for our network. Our model loss drops sharply at first and then reaches a plateau around iteration 600 and then begins to decrease linearly and converges to around 1.2. The loss of the model still decreased linearly when we cut off the training process.

#### 4.3.2. Selectivity Analysis of Convolutional Feature

The purpose of this paper is to try to combine the strong feature learning ability of CNN and the invariant nature of BoVW coding model. This section focuses on solving the problem of embedding BoW model in CNN structure and selects the hierarchical feature of CNN by reconstructing the image.

**Figure 10:**
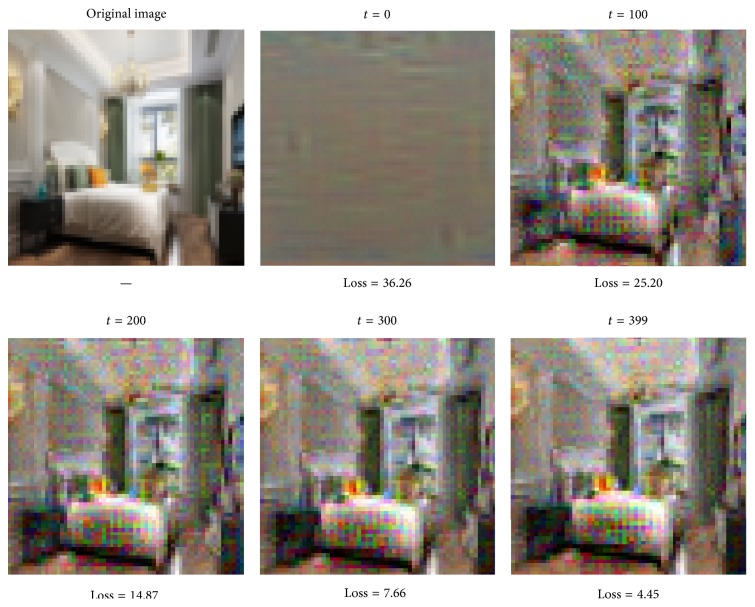
Reconstruct features from the third layer of the pretrained model.

**Figure 11:**
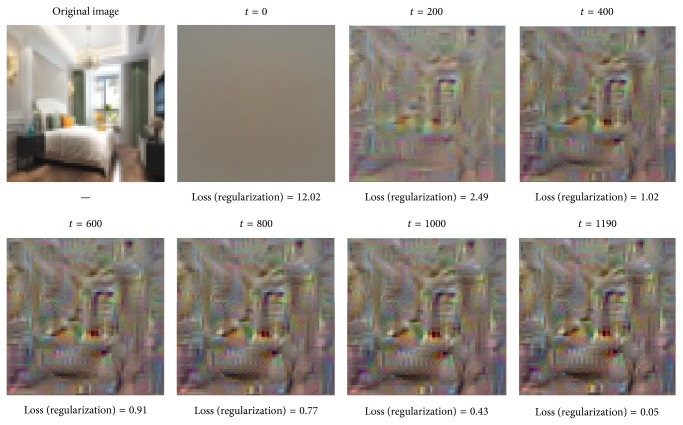
Reconstruct features from the fifth layer of the pretrained model.

Reconstructing images using features from deeper layers of the network tends to decide which layer of the convolutional layer will be embedded in the BoVW model. As Figure 10 shows, we reconstruct the features using the provided optimization parameters from the third convolutional layer of the pretrained model. In the cell below, we try to reconstruct the best image you can by inverting the features from the fifth layers in Figure 11. We will need to play with the hyperparameters to try and get a good result.

From the paper by Mahendran et al. [34, 37], we will see that reconstructions from deep features tend not to look much like the original image, so we should not expect the results to look like the reconstruction above. But we should be able to get an image that shows some discernable structure within 1000 iterations.

### 4.4. Parameter Settings

After obtaining the convolutional feature, we make full use of the invariance property of the BoVW model to obtain a more discriminative high-level distributed representation. This can not only avoid the gradient disappear in training and increase the identification of the characteristics of the middle convolution, but also increase the spatial structure information. After embedding into the BoVW model, the experiment encountered the following parameters.

#### 4.4.1. The Selection of *K* Values

**Figure 12:**
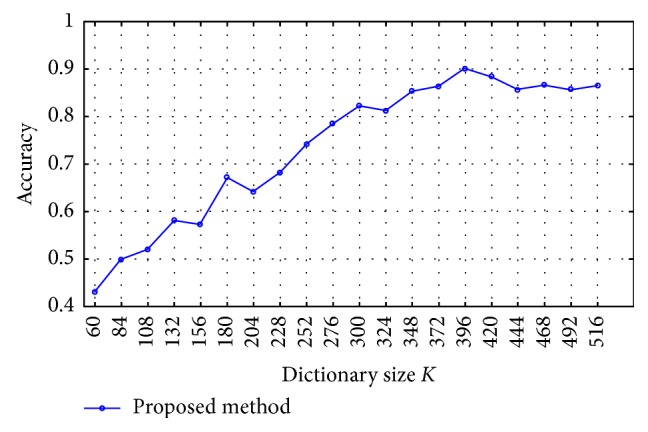
Comparison of different size *K* in our method.

An important parameter in the BoVW model is dictionary size *K*, which needs to be tuned. In order to determine their values, Figure 12 shows the classification accuracy versus dictionary size *K*. From this figure, we can find that when *K* = 396, our approach obtains the best performance (90.14%).

#### 4.4.2. Results of Different Kernel Functions

There are various options for this classifier, including GMM-type probabilistic models, SVMs, and neural networks. In this section, a simple SVM classifier [38] is employed in our method. The selection of kernel function is very important in the training process, because it determines the classification effect of the trained model. Next, we discuss the most suitable kernel function by experiment. This paper extracts six categories of images from the MIT indoor 67 data sets (bakery, classroom, elevator, museum, restaurant, and train station) to carry out classification experiments, and the experiment was divided into three parts: two classes (bakery, classroom), four-class classification (bakery, classroom, elevator, and museum), and six-class classification (bakery, classroom, classification elevator, museum, restaurant, and train station). Firstly, according to the previous chapter, the visual feature extraction, clustering, and expression are carried out, and the experimental results are the average of 10 experiments.  Table 1 gives the statistical results of SVM classification corresponding to different kernel functions.

Different kernel functions will be used to form different algorithms. The accuracy of these algorithms is greatly affected by the kernel function. Through the experimental comparison, we can see that the kernel function is the best choice and finally use the histogram intersection kernel (HIK) function for SVM classification.

### 4.5. Results and Discussion

#### 4.5.1. Computational Cost

Our CNN is implemented using TensorFlow [35]. With TensorFlow we can easily run our algorithm on a GPU to speed up the process without much optimization. Our experiments are performed on a PC with 3.4 GHz CPU and GeForce 980Ti GPU. We measure the processing time on images using our model of 50 kernels with 198 × 198 input size and test the model using the part of those strides that gives the state-of-the-art performance in the experiments on 15-scene dataset.  Table 2 shows the average processing time per image under different strides. Note that our implementation is not fully optimized. For example, the normalization process for each image is performed on the CPU in about 0.031 sec, which represents a significant portion of the total time. From Table 2 we can see that, with a sparser sampling pattern (stride greater than 64), real time processing can be achieved while maintaining state-of-the-art performance.

At the same time, as shown in Table 4, we also recorded the fine-tuning of different models in the same visual database running time. The experiments show that our method under the condition of consumption in a certain time achieved good classification effect.

#### 4.5.2. Performance on Different Datasets

After training our net for 100000 iterations, each iteration was composed by a random proportional subsample of 64 images across the entire dataset. In this section, we report results the performance on different datasets compared with previous studies. As shown in Table 3, in order to verify the classification performance of our method be more comprehensively, we compare our best results with the newly improved CNN method and other various state-of-the-art methods that have reported classification accuracy on 15-scene and MIT indoor datasets.

On the 15-scene dataset, our method achieves 90.1% accuracy, which exceeds the majority of existing methods. On the indoor-67 dataset, the two best reported accuracies are 63.18 [33] and 68.88 [25]. One used Fisher vector model and another extracted CNN activation for local patches at multiple scale levels. The accuracy of the activation features is rather close to the MOP-CNN [25]. However, these methods result in signature dimensionalities in the order of O (10^6^), whose dimensionality can be almost one order of magnitude higher than our characteristic dimension.

**Figure 13:**
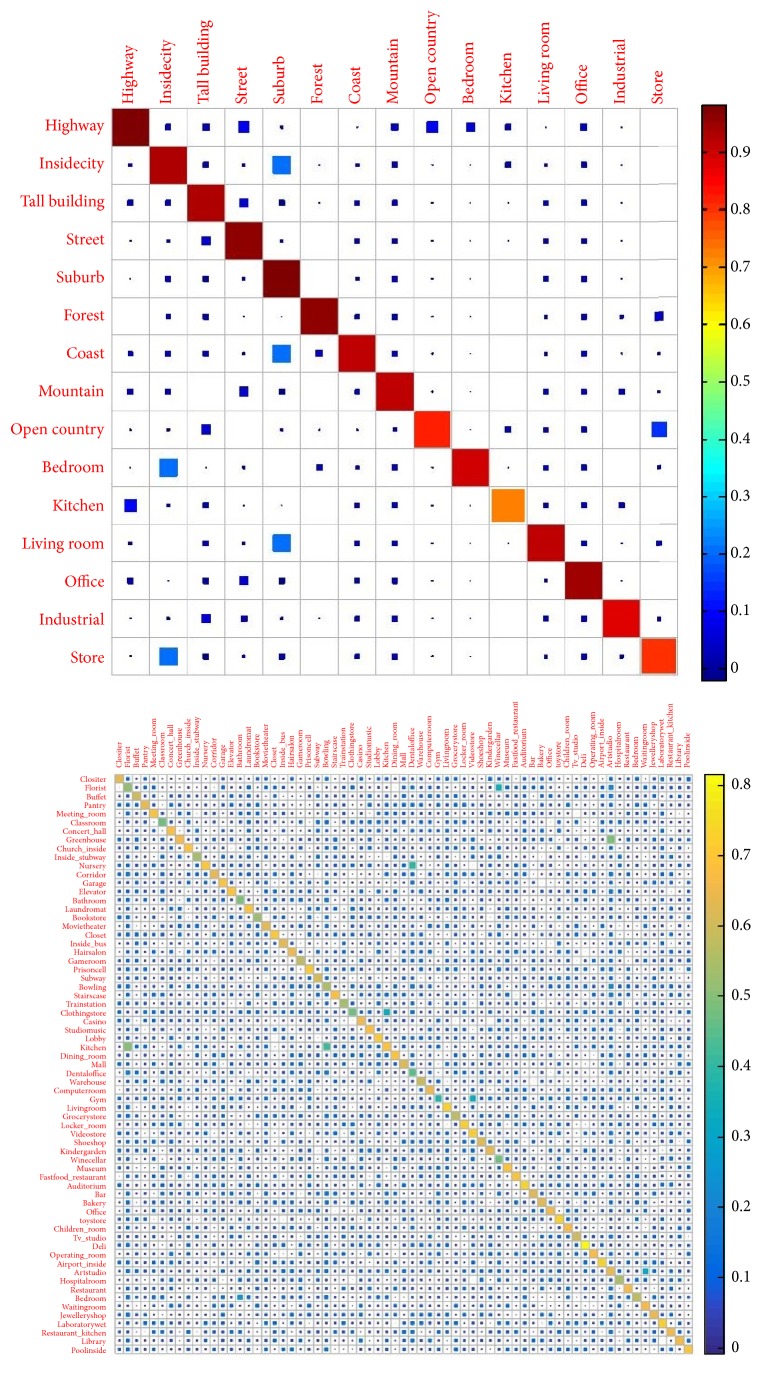
The visual predicted matrix of 15-scene and MIT indoor-67 datasets.

In addition, in order to further refine the performance evaluation results, confusion matrix method is usually used to evaluate the performance of classification model in different categories of scene image content. Among them, the element values on the diagonal of the confusion matrix reflect the accuracy of the visual word packet model for each scene image classification; the higher the accuracy, the better the performance. Based on the predicted category for each test case, we will now construct a confusion matrix. Entry (*i*, *j*) in this matrix would be the proportion of times a test image of ground truth category *i* was predicted to be category *j*. An identity matrix is the ideal case. The visual predicted matrixes for two different datasets are shown in Figure 13. Through this color confusion matrix, we can intuitively know the scope of the overall accuracy, as well as what kind of classification effect is better. For example, from the visual confusion matrix, through color changes and contrast, we easily recognize that suburb is very easy to recognize.

In order to evaluate our model training results, we choose another six methods to do a comparative experiment on the similar data set. We used the traditional two methods and also reproduced the method which uses BoVW model [39]. Simultaneously, we fine-tune the three famous and well-trained CNN models to make the model adaptable to the new classified data set.

Since each neural network model has the desired image input size, we adjust the image size in the same way so that it can be used in this network model and output the corresponding classification and annotation. To make the comparison process more fair, all the parameters of each super-model, including the right to reinitialize, gradient descent optimization algorithm, the weights learning rate decay and decline algorithm, and training time, test time is set to the same value. We also modeled the last layer of the output layer into the same BoVW model to compare the classification results. All experiments are repeated ten times with different randomly selected training and test images, and the average of per-class recognition rates is recorded for each run. The final result is reported as the mean and standard deviation of the results from the individual runs, as shown in Table 4.

#### 4.5.3. Visualized Table

**Figure 14:**
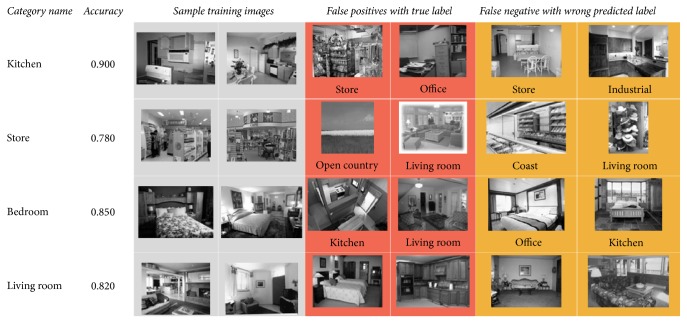
A visual table in fifteen-scene categories.

We create convenient and intuitive interface to visualize the classification results table. This table is with one row per category, with 3 columns, training examples, false positives, and false negatives. False positives are instances claimed as that category but belonging to another category; for example, in the “Kitchen” row an image was classified as “Kitchen” but is actually “Store.” This same image would be considered a false negative in the “Store” row, because it should have been claimed by the “Store” classifier but was not. False negatives are instances belonging to true category but with wrong predicted label. As shown in Figure 14, we show the first four lines of the table. From this table, we can intuitively see which scene images are marked with wrong labels, and which scene images have not been identified.

## 5. Conclusions

In this paper, we present a method for geographical scene classification, which is a generalized version of convolutional neural networks based on higher level of hierarchy. As objects are the parts that compose a scene, detectors tuned to the objects that are discriminant between scenes are learned in the inner layers of the network. The learned parts have been shown to perform well on the task of scene classification, where they improved a very solid bag of words. According to simulation results applied to fifteen-scene categories, MIT indoor-67 dataset, and 12-scene dataset, even with less number of trainable parameters in less time the proposed hierarchical structure could classify unseen samples with higher accuracy. In the future we will explore how to improve encoding technique to ensure that the CNN descriptors are embedded into a finite set of prototypical elements in order to classify geotagging images.

## Figures and Tables

**Table 1:** Classification accuracy of different kernel functions.

Name	2-class classification	4-class classification	6-class classification
Ploy	82.24%	79.20%	71.18%
RBF	87.57%	80.13%	73.68%
HIK	88.60%	84.41%	77.59%

**Table 2:** Time cost under different strides.

Stride	32	64	96	128
Time (sec)	0.102	0.036	0.023	0.018

**Table 3:** The classification accuracy of different methods on 15-scene and MIT indoor datasets.

Number	Method	MIT indoor (%)	15-scene (%)
1	SPM [4]	34.40	81.4
2	OTC [30]	47.33	—
3	LPR [31]	44.8	85.8
4	Discriminative patches ++ [32]	49.40	—
5	FV + bag of parts [33]	63.18	—
6	MOP-CNN [25]	68.88	—
7	Our approach	66.09	90.1

**Table 4:** The classification accuracy of different fine-tuning model on 12-scene datasets.

Number	Method	12-scene (%)	Time (h)
1	*K*Means + SVM	59.80	0.5
2	Sift + BoVW	61.02	0.5
3	Local–global feature BoVW [34]	60.23	1.2
4	Fine-tuning Cifar + BoVW	51.12	12
5	Fine-tuning Alexnet + BoVW	67.01 ± 1.22	28
6	Fine-tuning GoogLeNet + BoVW	68.21 ± 0.61	36
7	Our approach	75.12	23

## References

[1] Csurka G., Dance C. R., Fan L., Willamowski J., Bray C. (2004). Visual categorization with bags of keypoints. *Workshop on Statistical Learning in Computer Vision Eccv*.

[2] Song D., Tao D. (2010). Biologically inspired feature manifold for scene classification. *IEEE Transactions on Image Processing*.

[3] Quelhas P., Monay F., Odobez J.-M., Gatica-Perez D., Tuytelaars T., Van Gool L. Modeling scenes with local descriptors and latent aspects.

[4] Lazebnik S., Schmid C., Ponce J. Beyond bags of features: spatial pyramid matching for recognizing natural scene categories.

[5] Oliva A., Torralba A. (2001). Modeling the shape of the scene: a holistic representation of the spatial envelope. *International Journal of Computer Vision*.

[6] Zheng Y., Jiang Y., Xue X. (2012). Learning hybrid part filters for scene recognition. *Computer Vision—ECCV 2012*.

[7] Sun J., Ponce J. Learning discriminative part detectors for image classification and cosegmentation.

[8] Fukushima K. (1988). Neocognitron: a hierarchical neural network capable of visual pattern recognition. *Neural Networks*.

[9] LeCun Y., Bottou L., Bengio Y., Haffner P. (1998). Gradient-based learning applied to document recognition. *Proceedings of the IEEE*.

[10] An D. C., Meier U., Masci J. Flexible, high performance convolutional neural networks for image classification[C]// IJCAI.

[11] Simard P. Y., Steinkraus D., Platt J. C. Best practices for convolutional neural networks applied to visual document analysis.

[12] Szegedy C., Liu W., Jia Y. Going deeper with convolutions.

[13] Hinton E G., Srivastava N., Krizhevsky A. (2012). Improving neural networks by preventing co-adaptation of feature detectors. *Computer Science*.

[14] Zhou B., Garcia L A., Xiao J. (2015). Learning deep features for scene recognition using places database. *Advances in Neural Information Processing Systems (NIPS)*.

[15] Razavian A. S., Azizpour H., Sullivan J., Carlsson S. Cnn features off-the-shelf: an astounding baseline for recognition.

[16] Hu F., Xia G.-S., Hu J., Zhang L. (2015). Transferring deep convolutional neural networks for the scene classification of high-resolution remote sensing imagery. *Remote Sensing*.

[17] Xu Y., Mo T., Feng Q., Zhong P., Lai M., Chang E. I.-C. Deep learning of feature representation with multiple instance learning for medical image analysis.

[18] Zuo Z., Wang G., Shuai B., Zhao L., Yang Q., Jiang X. (2014). Learning discriminative and shareable features for scene classification. *Lecture Notes in Computer Science (including subseries Lecture Notes in Artificial Intelligence and Lecture Notes in Bioinformatics)*.

[19] Makantasis K., Karantzalos K., Doulamis A., Doulamis N. Deep supervised learning for hyperspectral data classification through convolutional neural networks.

[20] Hu W., Huang Y., Wei L., Zhang F., Li H. (2015). Deep convolutional neural networks for hyperspectral image classification. *Journal of Sensors*.

[21] Chen Y., Lin Z., Zhao X., Wang G., Gu Y. (2014). Deep learning-based classification of hyperspectral data. *IEEE Journal of Selected Topics in Applied Earth Observations and Remote Sensing*.

[22] Vakalopoulou M., Karantzalos K., Komodakis N., Paragios N. Building detection in very high resolution multispectral data with deep learning features.

[23] Merentitis A., Debes C. Automatic fusion and classification using random forests and features extracted with deep learning.

[24] Cimpoi M., Maji S., Vedaldi A. Deep filter banks for texture recognition and segmentation.

[25] Gong Y., Wang L., Guo R., Lazebnik S. (2014). Multi-scale orderless pooling of deep convolutional activation features. *Lecture Notes in Computer Science (including subseries Lecture Notes in Artificial Intelligence and Lecture Notes in Bioinformatics)*.

[26] Niu X.-X., Suen C. Y. (2012). A novel hybrid CNN-SVM classifier for recognizing handwritten digits. *Pattern Recognition*.

[27] He K., Zhang X., Ren S., Sun J. (2015). Spatial pyramid pooling in deep convolutional networks for visual recognition. *IEEE Transactions on Pattern Analysis and Machine Intelligence*.

[28] Li F. F., Perona P. A bayesian hierarchical model for learning natural scene categories.

[29] Quattoni A., Torralba A. Recognizing indoor scenes.

[30] Margolin R., Zelnik-Manor L., Tal A. (2014). OTC: A novel local descriptor for scene classification. *Lecture Notes in Computer Science (including subseries Lecture Notes in Artificial Intelligence and Lecture Notes in Bioinformatics)*.

[31] Sadeghi F., Tappen M. F. (2012). Latent pyramidal regions for recognizing scenes. *Lecture Notes in Computer Science (including subseries Lecture Notes in Artificial Intelligence and Lecture Notes in Bioinformatics)*.

[32] Singh S., Gupta A., Efros A. A. (2012). Unsupervised discovery of mid-level discriminative patches. *Lecture Notes in Computer Science (including subseries Lecture Notes in Artificial Intelligence and Lecture Notes in Bioinformatics)*.

[33] Juneja M., Vedaldi A., Jawahar C. V., Zisserman A. Blocks that shout: Distinctive parts for scene classification.

[34] Nguyen A., Yosinski J., Clune J. Deep neural networks are easily fooled: High confidence predictions for unrecognizable images.

[35] Abadi M., Agarwal A., Barham P., Brevdo E., Chen Z. (2015). TensorFlow: Large-scale machine learning on heterogeneous systems. https://www.tensorflow.org/.

[36] Alonso J., Chen Y. (2009). Receptive field. *Scholarpedia*.

[37] Mahendran A., Vedaldi A. Understanding deep image representations by inverting them.

[38] Chang C., Lin C J., LIBSVM. (2007). A library for support vector machines. *Acm Transactions on Intelligent Systems & Technology*.

[39] Zhu Q., Zhong Y., Zhao B., Xia G.-S., Zhang L. (2016). Bag-of-Visual-Words Scene Classifier with Local and Global Features for High Spatial Resolution Remote Sensing Imagery. *IEEE Geoscience and Remote Sensing Letters*.

